# Carbon content, carbon fixation yield and dissolved organic carbon release from diverse marine nitrifiers

**DOI:** 10.1002/lno.12252

**Published:** 2022-10-27

**Authors:** Barbara Bayer, Kelsey McBeain, Craig A. Carlson, Alyson E. Santoro

**Affiliations:** ^1^ Department of Ecology, Evolution and Marine Biology University of California Santa Barbara California; ^2^ Present address: Department of Microbiology and Ecosystem Science University of Vienna Vienna Austria; ^3^ Present address: Department of Oceanography University of Hawai'i at Manoa Honolulu Hawaii

## Abstract

Nitrifying microorganisms, including ammonia‐oxidizing archaea, ammonia‐oxidizing bacteria, and nitrite‐oxidizing bacteria, are the most abundant chemoautotrophs in the ocean and play an important role in the global carbon cycle by fixing dissolved inorganic carbon (DIC) into biomass. The release of organic compounds by these microbes is not well quantified, but may represent an as‐yet unaccounted source of dissolved organic carbon (DOC) available to marine food webs. Here, we provide measurements of cellular carbon and nitrogen quotas, DIC fixation yields and DOC release of 10 phylogenetically diverse marine nitrifiers. All investigated strains released DOC during growth, representing on average 5–15% of the fixed DIC. Changes in substrate concentration and temperature did not affect the proportion of fixed DIC released as DOC, but release rates varied between closely related species. Our results also indicate previous studies may have underestimated DIC fixation yields of marine nitrite oxidizers due to partial decoupling of nitrite oxidation from CO_2_ fixation, and due to lower observed yields in artificial compared to natural seawater medium. The results of this study provide critical values for biogeochemical models of the global carbon cycle, and help to further constrain the implications of nitrification‐fueled chemoautotrophy for marine food‐web functioning and the biological sequestration of carbon in the ocean.

A fraction of the carbon dioxide (CO_2_) that is captured by marine phytoplankton at the surface sinks to depth as dead organic material, supporting a deep ocean food‐web of both microbes and higher trophic levels (Hannides et al. 2013; Giering et al. 2014; Choy et al. 2015). Organic matter decomposition in the mesopelagic releases ammonium, a reduced form of nitrogen that can be used as an energy source by chemoautotrophic nitrifying archaea and bacteria to fuel dissolved inorganic carbon (DIC) fixation into biomass (Ward 2011). Chemoautotrophic production provides a new, labile, nonsinking source of particulate organic matter to the deep ocean which is otherwise dominated by refractory organic carbon (Reinthaler et al. 2010; Middelburg 2011), supporting the heterotrophic microbial community in the mesopelagic (Hansman et al. 2009; Baltar et al. 2010).

The main nitrifiers in the ocean are ammonia‐oxidizing archaea, which oxidize ammonia (NH_3_) to nitrite (NO_2_^−^), and nitrite‐oxidizing bacteria, which further oxidize NO_2_^−^ to nitrate (NO_3_^−^) (Ward 2011). These two steps are assumed to be tightly coupled, as NO_2_^−^ typically does not accumulate in oxic, open ocean waters with measurable nitrification, with the exception of the primary nitrite maximum at the base of the euphotic zone (Lomas and Lipschultz 2006; Santoro et al. 2013). Despite this tight coupling, ammonia‐oxidizing archaea are approximately six times more abundant than nitrite‐oxidizing Nitrospinae bacteria at a given location and sampling depth throughout the Pacific Ocean (Santoro et al. 2019), possibly owing to their smaller cell size compared to nitrite‐oxidizing bacteria (e.g., Watson and Waterbury 1971; Mueller et al. 2021; Qin et al. 2017), or as a result of the higher theoretical energy yield from ammonia compared to nitrite oxidation (Bock and Wagner 2013). Ammonia‐oxidizing bacteria are thought to play a minor role in global ocean nitrification due to their overall low abundances (Santoro et al. 2010; Buchwald et al. 2015; Tolar et al. 2016).

Despite the known difference in theoretical energy yield, there are many uncertainties regarding the organic carbon yield from ammonia vs. nitrite oxidation (hereinafter referred to as DIC fixation yield) and the contribution of both physiological groups to chemoautotrophic DIC fixation in the ocean. Cultures of ammonia‐oxidizing archaea have recently been shown to release dissolved organic carbon (DOC) during growth (Bayer et al. 2019a), yet this component of organic carbon is not captured by conventional methods measuring DIC incorporation into biomass. As such, the release of DOC by chemoautotrophs might represent an as‐yet unaccounted source of organic material in the deep ocean potentially fueling the microbial loop, with important implications for the marine carbon cycle. However, it remains unclear if DOC release is a phenomenon only observed under specific culture conditions, restricted to select strains of ammonia‐oxidizing archaea, or a common feature shared by diverse autotrophic nitrifiers under natural conditions.

Here, we report combined measurements of DIC fixation and DOC release of 10 phylogenetically diverse marine nitrifiers comprising two genera of ammonia‐oxidizing archaea (*Nitrosopumilus* and *Ca*. Nitrosopelagicus), one genus of ammonia‐oxidizing bacteria (*Nitrosomonas*) and three genera of nitrite‐oxidizing bacteria (*Nitrospina*, *Nitrospira*, and *Nitrococcus*). We further explored the effect of different culture conditions, including environmentally relevant conditions of low substrate concentration and temperature, on these measurements using two nitrifier strains isolated from the Pacific Ocean (*Nitrosopumilus* sp. CCS1 and *Nitrospina* sp. Nb‐3). The results of this study will inform ecological theoretical models to further constrain DIC fixation yields associated with nitrification in order to better understand the dynamics involved in the sequestration of carbon in the ocean.

## Methods

### Nitrifier culture sources

Ammonia‐oxidizing archaeal cultures used in this study were three axenic *Nitrosopumilus* strains and one *Nitrosopelagicus* enrichment culture. *Ca*. Nitrosopelagicus brevis U25 originates from a North Pacific Ocean water sample (Santoro and Casciotti 2011; Carini et al. 2018). The level of enrichment during the time of this study was > 90% and co‐cultured heterotrophic bacteria belonged to the genera *Erythrobacter* and *Gracilimonas* as previously described (Santoro and Casciotti 2011). *Nitrosopumilus* sp. CCS1 is a novel strain isolated from a seawater sample collected from the California Current system in the North Pacific Ocean (Santoro et al. unpublished). *Nitrosopumilus adriaticus* NF5 (= JCM 32270^T^ = NCIMB 15114^T^) and *Nitrosopumilus piranensis* D3C (= JCM 32271^T^ = DSM 106147^T^ = NCIMB 15115^T^) were isolated from the Northern Adriatic Sea and have been described in detail (Bayer et al. 2016, 2019*c*).

The four axenic nitrite‐oxidizing bacterial strains, *Nitrospina gracilis* Nb‐211, *Nitrospina* sp. Nb‐3, *Nitrococcus mobilis* Nb‐231, and *Nitrospira marina* Nb‐295, were obtained from the culture collection of John B. Waterbury and Frederica Valois at the Woods Hole Oceanographic Institution (WHOI). *N. gracilis* Nb‐211 was isolated from surface waters of the South Atlantic Ocean (Watson and Waterbury 1971), *N. mobilis* Nb‐231 was isolated from a surface water sample obtained from the South Pacific Ocean (Watson and Waterbury 1971) and *N. marina* Nb‐295 was isolated from a water sample collected at a depth of 206 m from the Gulf of Maine in the Atlantic Ocean (Watson et al. 1986). *Nitrospina* sp. Nb‐3 was isolated from the Pacific Ocean off the coast of Peru and has not yet been officially described (Watson and Waterbury, unpublished), however, its genome has recently been sequenced suggesting it is phylogenetically distinct from the species *N. gracilis* (Bayer et al. 2022).

Ammonia‐oxidizing bacteria used in this study, *Nitrosomonas marina* C‐25 and *Nitrosomonas* sp. C‐15 (also referred to as strain Nm51, [Koops et al. 1991]), were both obtained from the culture collection at WHOI and were revived from 60‐year‐old cryostocks. Strain C‐15 was isolated from surface water (1‐m depth) of the South Pacific Ocean off the Peruvian continental shelf (Watson and Mandel 1971) and strain C‐25 was isolated from surface waters of the South Atlantic Ocean (200 miles off the Amazon River mouth) (Watson and Mandel 1971).

### Culture conditions

*N. adriaticus* NF5, *N. piranensis* D3C, *N. marina* C‐25, and *Nitrosomonas* sp. C‐15 were grown in HEPES‐buffered artificial seawater medium containing 1 mmol L^−1^ NH_4_Cl, and *Ca*. Nitrosopelagicus brevis U25 was grown in natural seawater medium containing 50 *μ*mol L^−1^ NH_4_Cl. *N. gracilis* Nb‐211, *N. marina* Nb‐295, and *N. mobilis* Nb‐231 were grown in artificial seawater medium supplemented with 1 mmol L^−1^ NaNO_2_. *Nitrosopumilus* sp. CCS1 and *Nitrospina sp*. Nb‐3 were grown under multiple culture conditions as indicated in the Results and discussion. All strains were routinely grown in 60 mL polycarbonate bottles (Nalgene) containing 50 mL culture medium, and bottles were incubated at 25°C (with the exception of *Ca*. Nitrosopelagicus brevis, which was always incubated at 22°C) in the dark without agitation.

The artificial seawater medium contained 18.54 g L^−1^ NaCl, 4.7 g L^−1^ MgSO_4_·7H_2_O, 3.55 g L^−1^ MgCl_2_·6H_2_O, 1.03 g L^−1^ CaCl_2_·2H_2_O, 0.51 g L^−1^ KCl, 0.14 g L^−1^ NaHCO_3_. The natural seawater medium consisted of aged seawater collected from the Santa Barbara Channel (~ 10 m depth, 0.2‐*μ*m pore size filtered and autoclaved). Artificial and natural seawater were supplemented with 2.6 mg L^−1^ K_2_HPO_4_, 250 *μ*g L^−1^ FeNaEDTA, 30 *μ*g L^−1^ H_3_BO_3_, 20 *μ*g L^−1^ MnCl_2_·4H_2_O, 20 *μ*g L^−1^ CoCl_2_·6H_2_O, 24 *μ*g L^−1^ NiCl_2_·6H_2_O, 20 *μ*g L^−1^ CuCl_2_·2H_2_O, 144 *μ*g L^−1^ ZnSO_4_·7H_2_O, 24 *μ*g L^−1^ Na_2_MoO_4_·2H_2_O. The pH was adjusted to 7.8–8.0 with NaOH or HCl. Due to the pH decrease associated with ammonia oxidation, culture medium with high initial NH_4_^+^ concentrations (> 250 *μ*mol L^−1^) was buffered to pH 7.8 by addition of 10 mmol L^−1^ HEPES (54457; Sigma‐Aldrich). Ammonia‐oxidizing archaea cultures were supplemented with 50 U L^−1^ catalase (Cat. Nr. C9322; Sigma‐Aldrich) to reduce oxidative stress and nitrite‐oxidizing bacteria cultures were supplemented with 50 ng L^−1^ cyanocobalamin. To test the effect of reduced inorganic and organic nitrogen compounds on *Nitrospina* sp. Nb‐3, NH_4_Cl (50 *μ*mol L^−1^) or tryptone (150 mg L^−1^) were added to the culture medium.

NO_2_^−^ concentrations were measured using the Griess‐Ilosvay colorimetric method (Strickland and Parsons 1972) and enumeration of cells was performed on an Guava Easy‐Cyte flow cytometer (EMD Millipore) following SYBR Green staining as previously described (Bayer et al. 2021).

### Cellular carbon and nitrogen content measurements

To determine C : N ratios, between 100 and 500 mL of culture was filtered onto combusted (450°C, 4 h) glass fiber filters (Advantec, GF‐75, 25 mm; 0.3 *μ*m nominal pore size). Filters were acidified with HCl (10% vol/vol), dried (60°C, 24 h), and packed into tin capsules prior to being analyzed on a CHN elemental analyzer (Exeter Analytical, CEC 440HA). The instrument was calibrated with acetanilide following manufacturer protocols.

Cellular carbon (C) content was calculated using both, CHN elemental analyzer (only for large cells) and ^14^C‐DIC incorporation measurements (see below), divided by the number of newly produced cells. In addition, C content of the archaeal strain *Nitrosopumilus* sp. CCS1 was calculated from a dilution series of concentrated cells as described in White et al. (2019). Cells were concentrated using tangential flow filtration (Ultracell 30 kDa membrane; Pellicon; Millipore Sigma) and a dilution series of 1.1–5.6 × 10^11^ cells L^−1^ was constructed by resuspending cell concentrates in culture medium (Fig. S1). The total organic C content for each vial of the dilution series was directly measured by high temperature combustion using a modified Shimadzu TOC‐V as described in Carlson et al. (2010). C content per cell was calculated via linear regression of cell counts and elemental content over the dilution series, where the slope of a Model II least squares regression is considered the elemental content per cell (Fig. S1).

### Combined DIC fixation and DOC release measurements

DIC fixation was measured via the incorporation of [^14^C]‐bicarbonate as previously described (Herndl et al. 2005) with modifications. [^14^C]‐bicarbonate (specific activity 56 mCi mmol^−1^/2.072 × 10^9^ Bq mmol^−1^; PerkinElmer) was added to 5 mL of culture (between 10 and 60 *μ*Ci were added depending on the activity of the culture). Different incubation times were tested (see Results section) and all consecutive experiments were performed over the entire length of the growth curve. For every culture condition, at least three replicate live samples and one formaldehyde‐fixed blank (3% vol/vol) were incubated in temperature‐controlled incubators in the dark. Due to radiation safety procedures which preclude measurements of radioactive samples in general‐use equipment, parallel incubations without [^14^C]‐tracer additions were used to determine cell abundance and nitrite concentration (see above). Great care was taken to ensure the exact same culture conditions to reduce potential biological heterogeneity between replicates.

Incubations were terminated by adding formaldehyde (3% vol/vol) to 5 mL of sample. After 30–60 min, every sample was individually filtered onto 25 mm, 0.2 *μ*m pore size polycarbonate filters (Millipore) and rinsed with 0.5 mL of artificial seawater using a glass filtration set (Millipore). The individual filtrates (5.5 mL per sample) were collected and transferred to scintillation vials to determine the fraction of [^14^C]‐DOC. Excess [^14^C]‐bicarbonate from the filters was removed by exposing them to fumes of concentrated HCl (37%) for 24 h. The filters were transferred to scintillation vials and 10 mL of scintillation cocktail (Ultima Gold; PerkinElmer) was added. The filtrates were acidified to pH ∼ 2 with HCl (25%) as previously described (Marañón et al. 2004), and filtrates were kept for 24 h in open scintillation vials placed on an orbital shaker before 10 mL scintillation cocktail was added to each vial. Samples were shaken for ca. 30 s and incubated in the dark for at least 24 h prior to counting the disintegrations per minute (DPM) in a scintillation counter (Beckman Coulter LS6500) for 15 min.

Total radioactivity measurements were performed to verify added [^14^C]‐bicarbonate concentrations by pipetting 100 *μ*L of sample into scintillation vials containing 400 *μ*L beta‐phenylethylamine (to prevent outgassing of ^14^CO_2_). Scintillation cocktail was added, vials were shaken for ca. 30 s and immediately measured in the scintillation counter.

The resulting mean DPM of the samples were corrected for the DPM of the blank, converted into organic carbon fixed over time and corrected for the DIC concentration in the culture media.

DIC fixation rates were calculated using the following formula:

$$\left({\mathrm{DPM}}_{s}-{\mathrm{DPM}}_{b}\right)\times {\mathrm{DIC}}_{w}/\left({\mathrm{DPM}}_{\mathrm{tr}}\times \text{incubation time}\right),$$

where DPM are the disintegrations per minute measured in the scintillation counter, for the sample (s) and the blank (b). DIC_w_ denotes the dissolved inorganic carbon concentration in culture medium and DPM tracer (tr) is the DPM for the [^14^C]‐bicarbonate added to the incubations.

### DIC concentration measurements

Total alkalinity (TA) of unfixed natural and artificial seawater medium was measured via an open‐cell endpoint titration using a Mettler‐Toledo T5 autotitrator, and pH was measured spectrophotometrically using a Shimadzu UV‐1280 UV–VIS spectrophotometer as described previously (Dickson et al. 2007; Hoshijima and Hofmann 2019). DIC concentrations were calculated from TA and pH using the CO2SYS software (Pelletier 2007). To calculate DIC concentrations of HEPES‐buffered media, TA values were taken from unbuffered artificial seawater medium and the pH was remeasured after adding HEPES.

### Calculations of Gibbs free energy (Δ*G*)

The effective Gibbs free energy (Δ*G*) for ammonia and nitrite oxidation was calculated for the culture conditions in this study using the following formula:

$$\Delta G=\Delta G^{0}+\mathrm{RT}\;\ln\;Q,$$

where *R* is the ideal gas constant (8.314 J mol^−1^ K), *Q* is the reaction quotient, and *T* is the temperature in K. Δ*G* ^0^ values were obtained from Amend and Shock (2001).

*Q* was calculated based on the following measurements and estimates: NO_2_^−^ concentrations were measured directly (see above); [NO_3_^−^] and [NH_4_^+^] were estimated from the decrease or increase in [NO_2_^−^], respectively; NH_3_ concentrations were calculated based on [NH_4_^+^], pH of the culture medium, and the acid association constant (pKa = 9.4); and O_2_ concentrations were estimated to be 235 *μ*mol L^−1^ under completely oxic conditions during our incubations. A correction for ionic strength was applied according to Amend and LaRowe (2019). Calculations can be found in the Supporting Information (Table S1).

### Statistical analyses

Pairwise comparisons were performed with a two‐sided Mann–Whitney *U* test (pairwise.wilcox.test) using the R software environment (R Core Team 2013). *p* values were adjusted for multiple comparisons using the Benjamini–Hochberg correction (p.adjust.method = “fdr”) (Benjamini and Hochberg 1995).

## Results and discussion

### Elemental composition of cultured nitrifiers

We determined the cellular carbon (C) content of cultured isolates of ammonia‐oxidizing archaea, ammonia‐oxidizing bacteria, and nitrite‐oxidizing bacteria belonging to six different genera. The cellular C contents of ammonia‐oxidizing archaea were ~ 11–17 fg C cell^−1^ (Table 1), which is slightly higher than values reported for natural populations in the deep Atlantic Ocean (~ 8.39 fg cell^−1^, Herndl et al. 2005) and an enrichment culture from the Baltic Sea (9 fg cell^−1^, Berg et al. 2014), but much lower than values reported for ammonia‐oxidizing archaea from hypoxic shelf waters of the Gulf of Mexico (50 ± 16 fg cell^−1^, Kitzinger et al. 2020). All investigated marine ammonia‐ and nitrite‐oxidizing bacteria had higher cellular C quotas compared to archaeal nitrifiers (Table 1), with *Nitrospina* exhibiting the lowest (~ 28–55 fg C cell^−1^) and *Nitrococcus* the highest (~ 272–1207 fg C cell^−1^) values (Table 1). The C content of ammonia‐oxidizing archaea cells remained fairly constant during different growth phases, while C contents of all investigated nitrite oxidizer strains drastically decreased (~ 40–70%) from early exponential growth to stationary phase, which was supported by the observation of smaller cells in stationary compared to exponentially growing cultures (data not shown). Cell sizes of natural populations of Nitrospinae bacteria have been reported to be 4‐fold (Kitzinger et al. 2020) and 50‐fold (Pachiadaki et al. 2017) larger than cells of ammonia‐oxidizing archaea, potentially reflecting these variations in cell size and C content during different growth phases.

**Table 1:** Elemental stoichiometry of phylogenetically diverse cultured marine nitrifiers during different growth phases (early exponential, late exponential, stationary) including previously published values. C : N ratios were obtained during exponential growth phase. Cellular C content values are derived from DIC incorporation measurements if not stated otherwise.

Organism	C : N (mol mol^−1^)	Cellular content (fg C cell^−1^)			Ref.
		Early exponential	Late exponential	Stationary	
Ammonia‐oxidizing archaea					
*Ca*. Nitrosopelagicus brevis U25	n.d.	n.d.	10.8	n.d.	This study
*Nitrosopumilus sp*. CCS1	4.03 ± 0.32	11.8 ± 0.2	12.0 ± 2.0/12.5*	12.9 ± 2.0/16.3 ± 0.2^†^	This study
*Nitrosopumilus adriaticus* NF5	3.91	n.d.	16.7 ± 7.5	17.3 ± 2.3	This study, Bayer et al. (2019*c*)
*Nitrosopumilus piranensis* D3C	3.98	n.d.	16.3	17.2 ± 1.9	This study, Bayer et al. (2019*c*)
*Nitrosopumilus maritimus* NAOA6	5.8/5.9^‡^	n.d.	n.d.	34 ± 14/17 ± 6^‡^	Meador et al. (2020)
Ammonia‐oxidizing bacteria					
*Nitrosomonas* sp. C‐15	4.31 ± 0.11	n.d.	145.7 ± 11.1	115.2 ± 3.8	This study
*Nitrosomonas marina* C‐25	4.38 ± 0.14	n.d.	302.4 ± 10.0	159.7 ± 13.4	This study
*Nitrosomonas marina*	5.59–6.11^§^	241	139	133	Glover (1985)
*Nitrosococcus oceani*	3.58–4.95^§^	1115	961	919	Glover (1985)
Nitrite‐oxidizing bacteria					
*Nitrospina gracilis* Nb‐3	3.41 ± 0.05	50.8 ± 3.9	40.1 ± 2.5	28.4 ± 4.6	This study
*Nitrospina gracilis* Nb‐211	3.43 ± 0.18	54.9 ± 4.9^||^	n.d.	30.4 ± 3.4	This study
*Nitrospira marina* Nb‐295	4.22 ± 0.03	153.5 ± 18.1/155.2 ± 6.5^||^	69.5 ± 7.5	57.8 ± 6.2	This study
*Nitrococcus mobilis* Nb‐231	4.60 ± 0.13	994.6 ± 315.4/1206.6 ± 156.1^||^	442.9 ± 38.0	272.1 ± 60.7	This study
*Nitrococcus mobilis*	3.07–4.75^§^	1226	671	384	Glover (1985)

* Value obtained from TOC dilution series (see Methods section and Fig. S1).

^†^ Grown in HEPES‐buffered medium.

^‡^ Values obtained under phosphate‐replete and phosphate‐deplete conditions (P replete/P deplete).

^§^ Range of values obtained during different growth conditions.

^||^ Values obtained from CHN elemental analyzer measurements (see Methods section).

Due to the small cell sizes of ammonia‐oxidizing archaea and the potential to lose some cells via filtration through 0.2 *μ*m pore size filters, we compared the cellular C content of *Nitrosopumilus* sp. CCS1 to values obtained from a filtration‐independent method using a dilution series of concentrated cells (see Methods section). There was no difference between the cellular C content of exponentially growing cells of strain CCS1 when using either method (12.0 ± 2.0 vs. 12.5 fg cell^−1^, see Table 1). In addition, we quantified the amount of strain CCS1 cells passing through 0.2 *μ*m filter sizes via flow cytometry and found that 0.2 *μ*m polycarbonate filters had very high retention efficiency with only 0.14 ± 0.03% of cells not being recovered, further confirming that our results are not biased by the applied filtration procedure.

The molar C : N ratios of all investigated nitrifiers were in the range of 3.4–4.6 : 1 (Table 1), with the exception of previously published values of *Nitrosopumilus maritimus* NAOA6 (Meador et al. 2020) and two ammonia‐oxidizing bacteria strains (Glover 1985). The values observed are lower than average values of heterotrophic marine bacteria cultures (~ 5 : 1) including *Pelagibacter ubique* (~ 4.6 : 1) (White et al. 2019, and references therein), with *Nitrospina* cells exhibiting the lowest average C : N ratio (~ 3.4) of all cultured nitrifiers in our study (Table 1). These low cellular C : N ratios are surprising considering the observation of glycogen storage deposits in cells of *N. gracilis*, *N. mobilis*, and *N. marina* (Watson and Waterbury 1971; Watson et al. 1986), as well as polyhydroxbutyrate storage in *N. mobilis* (Watson and Waterbury 1971). Cellular N contents in our study might be underestimated as measurements were performed on acidified samples, which could lead to partial hydrolyzation of proteins and amino acids. However, C : N ratios obtained from non‐acidified cell pellets of *N. adriaticus* and *N. piranensis* (Bayer et al. 2019*c*) are comparable to those obtained for *Nitrosopumilus* sp. CCS1 in this study (Table 1), suggesting a negligible bias for at least some of the studied species.

### DIC fixation yields of marine nitrifiers

We conducted combined measurements of DIC fixation, DOC release and ammonia/nitrite oxidation rates of 10 nitrifier cultures. The biological variability of NO_2_^−^ and DIC fixation measurements between replicate bottles across all nitrifier strains and culture conditions was 5.2 ± 3.7% and 4.8 ± 3.2%, respectively. Here, we use the term “DIC fixation yield” to describe the number of moles of inorganic carbon (CO_2_ or HCO_3_^−^) that are fixed for every mole of N (NH_3_ or NO_2_^−^) oxidized, including the proportion that is released/lost as DOC.

**Fig. 1:**
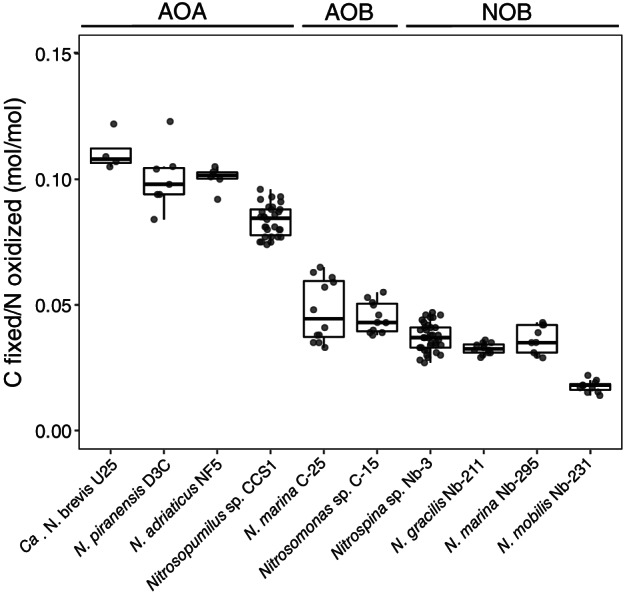
Comparison of DIC fixation yields of 10 different phylogenetically diverse marine nitrifiers. Plotted values include both, the fraction of C incorporated into biomass and the fraction of C released as DOC. For nitrite‐oxidizing bacteria, only measurements conducted over the entire length of the growth curve (until stationary phase) are shown (see Fig. 2). Values obtained from cultures grown under different conditions (see Figs. 3, fig. S3) are included in this plot. Cell‐normalized DIC fixation rates are reported in Table S2.

Marine ammonia‐oxidizing archaea, including three axenic *Nitrosopumilus* strains and one *Ca*. Nitrosopelagicus enrichment culture, exhibited the highest DIC fixation yields (mean ± SD = 0.091 ± 0.012, *n* = 47) in our study, which were on average ~ 2 times higher than those of marine ammonia‐oxidizing bacteria (mean ± SD = 0.047 ± 0.010, *n* = 23) (Fig. 1). Ammonia‐oxidizing archaea encode the 3‐hydroxypropionate/4‐hydroxybutyrate (3‐HP/4‐HB) cycle for DIC fixation (Walker et al. 2010), which is suggested to be the most energy‐efficient aerobic autotrophic DIC fixation pathway (Könneke et al. 2014). In contrast, ammonia‐oxidizing bacteria use the Calvin–Benson–Bassham (CBB) cycle (Utåker et al. 2002; Stein et al. 2007), which has a higher ATP requirement and an estimated 20% loss of fixed DIC due to the oxygenase side‐reaction of ribulose‐1,5‐bisphosphate carboxylase/oxygenase (Berg 2011). DIC fixation yields of two *Nitrosopumilus* strains were recently reported to be up to 10 times higher (0.18–1.2, Meador et al. 2020) compared to values in our study and previously published values of *N. adriaticus* NF5 (0.1, Bayer et al. 2019*c*) and a *Nitrosarchaeum* enrichment culture (0.1, Berg et al. 2014). However, such high values would require unrealistically high ATP yields (up to 2.4 mol ATP per mole NH_3_ oxidized) compared to reported estimates of 0.15–0.28 ATP/NH_3_ (mol/mol) (Li et al. 2018).

**Fig. 2:**
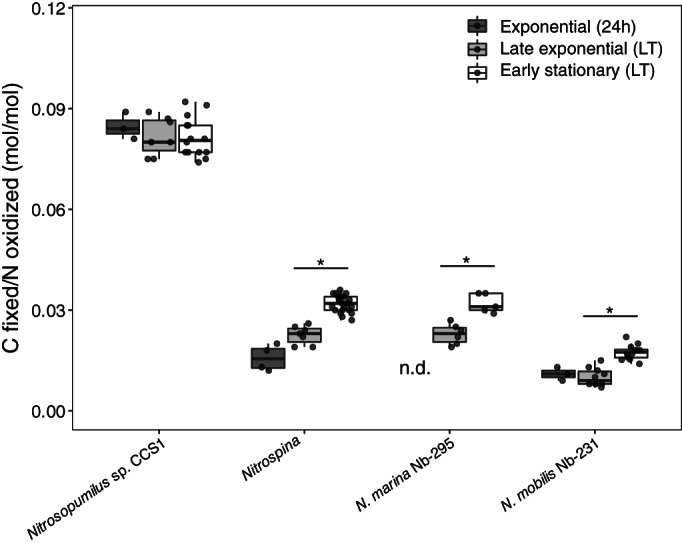
Comparison of DIC fixation yields obtained from 24 h radiotracer incubations during exponential growth, and long‐term (LT) radiotracer incubations carried out until either late exponential growth or early stationary phase. Measurements of both *Nitrospina* strains (Nb‐3 and Nb‐211) were combined in this plot. Statistical significance (adj. *p* < 0.01) of within‐condition comparisons are indicated by an asterisk (*). Statistical results of all pairwise comparisons are reported in Table S3. Representative growth curves can be found in the Supporting Information (Fig. S2).

DIC fixation yields of marine nitrite‐oxidizing bacteria (*Nitrospina*/*Nitrospira*: mean ± SD = 0.036 ± 0.005, *n* = 47; *Nitrococcus*: mean ± SD = 0.018 ± 0.002, *n* = 11) were lower compared to those of ammonia oxidizers (Fig. 1). *N. mobilis*, which uses the CBB cycle for DIC fixation (Füssel et al. 2017) had ~ 2 times lower DIC fixation yields compared to *Nitrospina* and *Nitrospira* which use a O_2_‐tolerant version of the reverse TCA cycle (Lücker et al. 2010, 2013). Zhang et al. (2020) measured ~ 1.7‐times lower DIC fixation yields of *N. gracilis* 3/211 and a terrestrial *Nitrospira* isolate compared to values in our study. We observed that radiotracer incubations conducted over the entire length of the growth curve (until early stationary phase, see Fig. S2) resulted in ~ 1.4–1.7‐times higher DIC fixation yields of nitrite oxidizers compared to incubations conducted until late exponential growth (when NO_2_^−^ was completely oxidized) (Fig. 2), suggesting that, in contrast to *Nitrosopumilus* sp. CCS1 where ammonia oxidation and DIC fixation were tightly coupled, nitrite oxidation might be partly decoupled from DIC fixation in nitrite‐oxidizing bacteria. This observation was also supported by an increase of cell numbers after NO_2_^−^ was completely depleted (Fig. S2). While incubation times < 72 h are typically favored over longer times for environmental measurements to avoid cross‐feeding of reaction products, our results indicate that DIC fixation yields of nitrite oxidizers might be underestimated using these established protocols (Fig. 2).

**Fig. 3:**
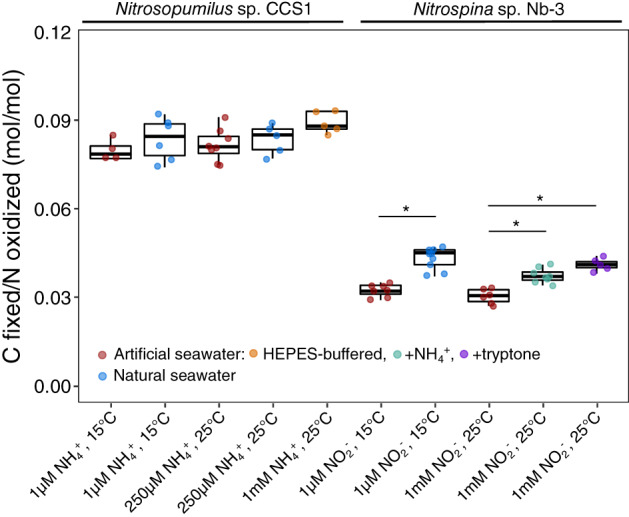
DIC fixation yields of *Nitrosopumilus* sp. CCS1 and *Nitrospina* sp. Nb‐3 under different culture conditions (substrate concentrations: 1 *μ*mol L^−1^, 250 *μ*mol L^−1^, 1 mmol L^−1^; temperature: 15°C, 25°C) and culture media (natural seawater, artificial seawater, HEPES‐buffered artificial seawater). Plotted values include both, the fraction of C incorporated into biomass and the fraction of C released as DOC. Ammonium (50 *μ*mol L^−1^) or tryptone (150 mg L^−1^) served as additional, reduced nitrogen source for *Nitrospina* sp. Nb‐3. Statistical significance (adj. *p* < 0.01) of within‐condition comparisons are indicated by an asterisk (*). Statistical results of all pairwise comparisons are reported in Table S3.

We further explored the effect of multiple culture conditions, including environmentally relevant conditions of low substrate concentrations (1 *μ*mol L^−1^) and low temperature (15°C), on DIC fixation yields of *Nitrosopumilus* sp. CCS1 and *Nitrospina* sp. Nb‐3. We observed that *Nitrospina* sp. Nb‐3 was ~ 1.4 times more efficient in converting energy to growth when grown in natural seawater compared to artificial seawater medium, which was not observed for *Nitrosopumilus* sp. CCS1 (Fig. 3). We hypothesize that reduced N compounds present in natural seawater (ammonium and/or organic N compounds) might be responsible for the observed differences due to the metabolic costs of six reduced ferredoxins associated with assimilatory NO_2_^−^ reduction in *Nitrospina* and *Nitrospira* (Lücker et al. 2013; Bayer et al. 2021). Those reduced ferredoxins could instead be used as electron donors for DIC fixation via the rTCA cycle (Berg 2011). Additions of ammonium or tryptone to artificial seawater medium likewise resulted in significantly higher DIC fixation yields (Figs. 3, S3), corroborating this hypothesis. Environmental populations of *Nitrospinae* have previously been shown to favor ammonium and the organic N sources urea and cyanate over nitrite (Kitzinger et al. 2020). Our data suggest that in addition to urea and cyanate, marine nitrite‐oxidizing bacteria can assimilate more complex organic N sources such as peptides and/or amino acids thereby saving energy that can instead be invested in C assimilation. We cannot exclude the possibility that some of the organic C present in natural seawater or added via tryptone might also be assimilated, however, the observed higher net DIC fixation yields suggest that organic C assimilation plays—if at all—a very minor role in marine nitrite oxidizers. Furthermore, we observed slightly higher DIC fixation yields of *Nitrosopumilus* sp. CCS1 in HEPES‐buffered artificial seawater compared to unbuffered culture medium (Fig. 3), which coincided with higher cellular C quota (Table 1). While we cannot explain these observations, the differences in DIC fixation yield did not seem to be caused by variations in pH, which remained constant in unbuffered culture medium containing low substrate concentrations (1 *μ*mol L^−1^ NH_4_^+^).

The theoretical Gibbs free energy release (Δ*G*) estimated for conditions in our study was 3.6 times higher for ammonia compared to nitrite oxidation (Table 2), yet DIC fixation yields of *Nitrosopumilus* sp. CCS1 and *Ca*. Nitrosopelagicus brevis U25 (Table 2) were only 2–2.6 times higher compared to *Nitrospina* sp. Nb‐3. Similar observations were made by Kitzinger et al. (2020) who reported that Nitrospinae bacteria in low O_2_ waters of the Gulf of Mexico are more efficient in translating energy gained from nitrite oxidation to C assimilation than ammonia‐oxidizing archaea are in translating energy gained from ammonia oxidation. Thermodynamic properties and the efficiency of the DIC fixation pathway itself can contribute to realized energy yields. Additional factors include the requirement of four out of six generated electrons by ammonia monooxygenase to reduce molecular oxygen in ammonia oxidizers (Stahl and de la Torre 2012; Caranto and Lancaster 2018). When considering that a maximum of 53.8% of the energy released from catabolism are available to ammonia oxidizers for growth (González‐Cabaleiro et al. 2019), ammonia‐oxidizing archaea are estimated to have slightly higher DIC fixation efficiencies compared to nitrite‐oxidizing bacteria encoding the rTCA cycle (Table 2). While oxygen protection likely increases the energy demands of the rTCA cycle (Berg 2011), our results indicate that the cycle might also be highly efficient under oxic conditions that are found in most regions of the global ocean.

**Table 2:** Thermodynamic considerations and comparison of DIC fixation efficiencies and biomass yields of marine ammonia‐oxidizing archaea and nitrite‐oxidizing bacteria grown under environmentally relevant conditions (substrate concentration: 1 *μ*mol L^−1^; temperature: 15°C) in artificial and natural seawater medium. Gibbs free energy calculations for NH_3_ oxidation and NO_2_^−^ oxidation can be found in Table S1.

	*Ca*. Nitrosopelagicus U25*	*Nitrosopumilus* sp. CCS1		*Nitrospina* sp. Nb‐3	
Culture medium	Natural seawater	Artificial seawater	Natural seawater	Artificial seawater	Natural seawater
Gibbs free energy (kJ mol^−1^)	280/151^†^	276/149^†^	276/149^†^	77	77
DIC fixation yield (mol mol^−1^)	0.111 ± 0.008	0.080 ± 0.004	0.085 ± 0.008	0.032 ± 0.002	0.043 ± 0.004
DIC fixation efficiency (*μ*mol C kJ^−1^)	396 ± 29/735 ± 53^†^	290 ± 15/537 ± 27^†^	308 ± 29/570 ± 54^†^	416 ± 26	558 ± 52
Biomass yield^‡^ (gBio gN^−1^)	0.187 ± 0.019	0.135 ± 0.010	0.143 ± 0.019	0.056 ± 0.005	0.076 ± 0.010

* *Ca*. Nitrosopelagicus U25 was grown at 22°C with initial substrate concentrations of 50 *μ*mol L^−1^.

^†^ When considering 53.8% of the energy released is available for growth according to González‐Cabaleiro et al. (2019).

^‡^ The average chemical formula of bacterial biomass (CH_1.7_O_0.4_N_0.2_, Popovic 2019) was adjusted using the C : N ratios from Table 1 (ammonia‐oxidizing archaea: CH_1.7_O_0.4_N_0.25_; *Nitrospina*: CH_1.7_O_0.4_N_0.29_).

Multiple studies have used estimates of DIC fixation yields to infer DIC fixation rates associated with nitrification in diverse marine and estuarine environments (e.g., Dore and Karl 1996; Lam et al. 2004; Lee et al. 2015), and a value of 0.1 for archaeal ammonia oxidation has widely been used in the literature (Wuchter et al. 2006; Reinthaler et al. 2010; Middelburg 2011) without direct experimental evidence. Previous measurements of DIC fixation yields were mainly derived from cultures of ammonia and nitrite oxidizers that are not representative for the majority of nitrifiers found in marine environments and were highly variable (ammonia‐oxidizing bacteria: 0.033–0.130; nitrite‐oxidizing bacteria: 0.013–0.031; Prosser 1990, and references therein). The variations in DIC fixation yields we observe for marine nitrifiers across different species and culture conditions are comparably low within ammonia‐oxidizing archaea (mean ± SD = 0.091 ± 0.012; *n* = 47) and *Nitrospina*/*Nitrospira* (mean ± SD = 0.036 ± 0.005; *n* = 56), suggesting that these values are more constrained than previous estimates and particularly useful for modeling approaches in marine systems.

### DOC release by chemolithoautotophs

**Fig. 4:**
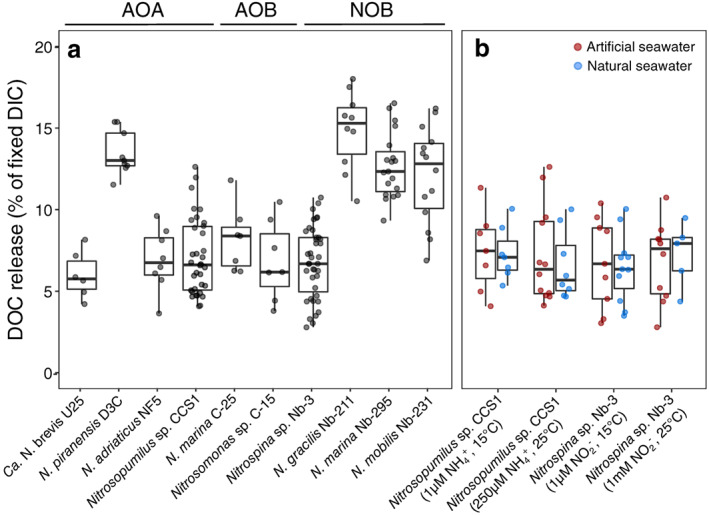
DOC release by marine nitrifiers as a fraction of fixed DIC. (**a**) Comparison of DOC release by 10 different phylogenetically diverse marine nitrifiers. Values obtained from cultures grown under different conditions (see panel **b**) are included in this plot. DOC release by *Ca*. N. brevis might be underestimated due to the presence of heterotrophic bacteria that could take up some of the released DOC. (**b**) Comparison of DOC release by *Nitrosopumilus* sp. CCS1 and *Nitrospina* sp. Nb‐3 grown under different culture conditions (substrate concentrations: 1 *μ*mol L^−1^, 250 *μ*mol L^−1^, 1 mmol L^−1^; temperature: 15°C, 25°C) in artificial or natural seawater medium. Statistical results of all pairwise comparisons are reported in Table S3.

We measured DOC release rates of 10 nitrifier cultures and tested how different culture conditions affected the amount of DOC released in proportion to the amount of fixed DIC. All investigated strains released DOC during exponential growth, and DOC release ceased when cultures reached stationary phase (as determined by comparing the total amount of released DOC until late exponential vs stationary phase, see Fig. S4), suggesting that DOC release is a feature of metabolically active nitrifiers. This is in agreement with earlier observations of amino acid release by exponentially growing *Nitrosopumilus* cells (Bayer et al. 2019*a*). The amount of chemoautotrophically fixed DIC that was released as DOC by nitrifiers made up on average ~ 5–15% (Fig. 4a). This is within the range observed for phytoplankton, which released 2–10% and 4–42% of their photosynthetically fixed DIC in culture and environmental studies, respectively (Carlson 2002, and references therein). To assess the potential stimulation of DOC release during the 30–60 min of formaldehyde fixation (see Methods section), we compared the fraction of fixed DIC released as DOC between 24 h and long‐term (7‐ and 10‐day long) incubations (Fig. S5). If DOC release was occurring during the fixation period, it would make up a larger fraction of the total DOC release in 24 h compared to long‐term incubations. However, we did not observe any significant differences between incubation times (Fig. S5), suggesting that formaldehyde fixation did not bias our results.

DOC release varied between closely related species (Fig. 4a). *N. piranensis* released more DOC compared to the two other investigated *Nitrosopumilus* species, which is in agreement with Bayer et al. (2019*b*) who reported higher amino acid release rates of *N. piranensis* compared to *N. adriaticus*. Differences in the amount of released DOC have also been recently reported between the closely related aquarium strain *N. maritimus* SCM1 (9–19% of fixed DIC) and the environmental strain *N. maritimus* NAOA6 (5% of fixed DIC) (Meador et al. 2020). Within nitrite oxidizers, *Nitrospina* sp. Nb‐3 consistently released less DOC compared to *N. gracilis* Nb‐211 and the two phylogenetically more distantly related species *N. marina* and *N. mobilis*.

The fraction of released DOC remained constant across different culture conditions including environmentally relevant conditions of low substrate concentration (1 *μ*mol L^−1^) and at low temperature (15°C) in natural seawater (Fig. 4b). This suggests that DOC release is not an artifact of unrealistic culture conditions but likely a feature exhibited by nitrifier populations in the environment. However, given the differences in DOC release between closely related cultured species (Fig. 4a) and the greater diversity of nitrifiers observed in the ocean, it is possible that DOC release might differ in more complex natural environments, particularly of marine nitrite oxidizers for which environmentally relevant clades escaped cultivation thus far (Pachiadaki et al. 2017). In addition, in situ pressure conditions could further affect DOC release by nitrifiers in nature. While the composition of DOM released by bacterial nitrifiers is currently unknown, a fraction of the DOM released by ammonia‐oxidizing archaea has been shown to consist of labile compounds, such as amino acids, thymidine, and B vitamins, that can be limiting for heterotrophic microbes in open ocean waters (Bayer et al. 2019*a*).

## Conclusions

Our results suggest that DIC fixation yields of marine nitrite oxidizers might be underestimated by conventional < 72 h‐long tracer incubations, due to a partial decoupling between NO_2_^−^ oxidation and C assimilation over short timescales. In addition, DIC fixation yields of *Nitrospina* were positively affected by the presence of ammonium or complex organic N compounds, which might influence metabolic interactions with ammonia oxidizers and/or heterotrophic prokaryotes in the environment, suggesting a potentially underappreciated role for competition in the N cycle (Santoro 2016).

DIC fixation yields of marine nitrifiers obtained in our study will help to further constrain the relationship between C and N fluxes in the nitrification process and inform theoretical models about how to connect observations at microscale to regional and global scales. Using a mean global value of organic C export from the euphotic zone of ~ 6 Pg C yr^−1^ (Siegel et al. 2014) and a mean C : N ratio of sinking marine particles (at the surface) of ~ 7.1 (Schneider et al. 2003), we estimate that the resulting global ocean organic N export of ~ 0.85 Pg yr^−1^ could fuel up to 0.13 Pg C y^−1^ of chemoautotrophic DIC fixation (0.094 Pg C yr^−1^ by ammonia‐oxidizing archaea and 0.037 Pg C yr^−1^ by nitrite oxidizers) in the dark ocean, which is in the lower range of previous estimates (0.15–1.4 Pg C yr^−1^, see Table S4, and references therein). Furthermore, we show that nitrifiers release significant amounts of DOC under environmentally relevant conditions, equating to fluxes of 0.006–0.02 Pg C yr^−1^ of fixed DIC released as DOC. Elucidating the lability and fate of the DOM released by nitrifiers will be crucial to understand its implications for the marine carbon cycle.

## Conflict of Interest

None declared.

## Supporting information


**Table S2.** Comparison of cell‐normalized DIC fixation rates of diverse marine cultured nitrifiers and from the open ocean. The range of values obtained from measurements of cultures during different growth phases is shown.
**Table S3.** Results of statistical analyses (see Materials and methods section in the main text). Significant adjusted *p* values are displayed in bold (a) Pairwise comparisons of DIC fixation yields of marine nitrifiers obtained under different growth phases (EXP, exponential; STAT, stationary) and incubation times (24 h; LT, long‐term). (b) Pairwise comparison of DIC fixation yields of Nitrosopumilus sp. CCS1 grown in different culture medium (ASW, artificial seawater; NSW, natural seawater; ASW‐HEPES, HEPES‐buffered artificial seawater). (c) Pairwise comparisons of DIC fixation yields of marine NOB grown in different culture medium (ASW, artificial seawater; NSW, natural seawater) with additions of ammonium (+ NH_4_^+^) or tryptone (+ trp) and at different temperature (15°C, 25°C) and NO_2_^−^ concentrations (1 μM, 1 mM NO_2_^−^).
**Table S4.** Comparison of estimates of global dark ocean DIC fixation fueled by ammonia and nitrite oxidation.
**Fig. S1.** Dilution series of concentrated cells to determine the cellular C content of Nitrosopumilus sp. CCS1.
**Fig. S2.** Nitrite and cell concentrations of Nitrosopumilus sp. CCS1 (A), Nitrospina sp. Nb‐3 (B) and Nitrococcus mobilis Nb‐231 (C).
**Fig. S3.** DIC fixation yield of N. marina Nb‐295 grown in artificial seawater medium with and without addition of tryptone.
**Fig. S4.** DOC release (as a proportion of fixed DIC) of marine nitrifiers during different growth phases.
**Fig. S5.** DOC release (as a proportion of fixed DIC) of Nitrosopumilus sp. CCS1 and Nitrospina sp. Nb‐3 at varying incubation times.

**Table S1.** Gibbs Free Energy calculations of ammonia and nitrite oxidation (provided as separate excel sheet)

## Data Availability

Data and metadata are available at the Biological and Chemical Oceanography Data Management office (BCO‐DMO) under project 806565.
