# Supplementary material for: Carbon content, carbon fixation yield and dissolved organic carbon release from diverse marine nitrifiers
Source: Limnol Oceanogr. 2022 Oct 27;68(1):84–96. doi: 10.1002/lno.12252 (PMC10092583; doi:10.1002/lno.12252)
Supplement: Supplementary file 1 — Table S2. Comparison of cell‐normalized DIC fixation rates of diverse marine cultured nitrifiers and from the open ocean. The range of values obtained from measurements of cultures during different growth phases is shown. Table S3. Results of statistical analyses (see Materials and methods section in the main text). Significant adjusted p values are displayed in bold (a) Pairwise comparisons of DIC fixation yields of marine nitrifiers obtained under different growth phases (EXP, exponential; STAT, stationary) and incubation times (24 h; LT, long‐term). (b) Pairwise comparison of DIC fixation yields of Nitrosopumilus sp. CCS1 grown in different culture medium (ASW, artificial seawater; NSW, natural seawater; ASW‐HEPES, HEPES‐buffered artificial seawater). (c) Pairwise comparisons of DIC fixation yields of marine NOB grown in different culture medium (ASW, artificial seawater; NSW, natural seawater) with additions of ammonium (+ NH4 +) or tryptone (+ trp) and at different temperature (15°C, 25°C) and NO2 − concentrations (1 μM, 1 mM NO2 −). Table S4. Comparison of estimates of global dark ocean DIC fixation fueled by ammonia and nitrite oxidation. Fig. S1. Dilution series of concentrated cells to determine the cellular C content of Nitrosopumilus sp. CCS1. Fig. S2. Nitrite and cell concentrations of Nitrosopumilus sp. CCS1 (A), Nitrospina sp. Nb‐3 (B) and Nitrococcus mobilis Nb‐231 (C). Fig. S3. DIC fixation yield of N. marina Nb‐295 grown in artificial seawater medium with and without addition of tryptone. Fig. S4. DOC release (as a proportion of fixed DIC) of marine nitrifiers during different growth phases. Fig. S5. DOC release (as a proportion of fixed DIC) of Nitrosopumilus sp. CCS1 and Nitrospina sp. Nb‐3 at varying incubation times. [file LNO-68-84-s001.pdf]

Supporting Information

Carbon content, carbon fixation yield and dissolved organic carbon release from diverse marine nitrifiers

Barbara Bayer[1,2*], Kelsey McBeain[1,3], Craig A. Carlson[1], and Alyson E. Santoro[1]

[1] Department of Ecology, Evolution and Marine Biology, University of California, Santa Barbara, CA, USA

[2] Current address: Department of Microbiology and Ecosystem Science, University of Vienna, Vienna, Austria

[3] Current address: Department of Oceanography, University of Hawai'i at Manoa, Honolulu, HI, USA

*Correspondence: barbara.bayer@univie.ac.at

Supporting Tables and Figures

Table S1. Gibbs Free Energy calculations of ammonia and nitrite oxidation (provided as separate excel sheet)

Table S2. Comparison of cell-normalized DIC fixation rates of diverse marine cultured nitrifiers and from the open ocean. The range of values obtained from measurements of cultures during different growth phases is shown.

Nitrifier strain	DIC fixation rate (fmol C $cell^{-1}$ d^{-1})	Reference
**Ammonia-oxidizing archaea**		
Ca. Nitrosopelagicus brevis U25	0.04-0.17	this study
Nitrosopumilus sp. CCS1	0.04-0.61	this study
Nitrosopumilus adriaticus NF5	0.09-0.80	this study
Nitrosopumilus piranensis D3C	0.05-0.82	this study
Open ocean ammonia-oxidizing archaea	0.002-0.10	Varela et al. 2011
**Nitrite-oxidizing bacteria**		
Nitrospina gracilis Nb-211	0.33-0.73	this study
Nitrospina sp. Nb-3	0.17-1.54	this study
Nitrospira marina Nb-295	0.13-0.96	this study
Nitrococcus mobilis Nb-231	1.76-9.12	this study
Open ocean Nitrospinae	0.002-0.73	Pachiadaki et al. 2017

Table S3. Results of statistical analyses (see Materials and Methods section in the main text). Significant adjusted *P*-values are displayed in bold **a)** Pairwise comparisons of DIC fixation yields of marine nitrifiers obtained under different growth phases (EXP, exponential; STAT, stationary) and incubation times (24h; LT, long-term). **b)** Pairwise comparison of DIC fixation yields of *Nitrosopumilus* sp. CCS1 grown in different culture medium (ASW, artificial seawater; NSW, natural seawater; ASW-HEPES, HEPES-buffered artificial seawater). **c)** Pairwise comparisons of DIC fixation yields of marine NOB grown in different culture medium (ASW, artificial seawater; NSW, natural seawater) with additions of ammonium ($+NH_4^+$) or tryptone (+trp) and at different temperature (15°C, 25°C) and NO_2^- concentrations (1 µM, 1 mM NO_2^-).

a)

	Nitrosopumilus sp. CCS1		*Nitrospina* sp. Nb-3		*N. mobilis* Nb-231		*N. marina* Nb-295	
	EXP (24h)	EXP (LT)	EXP (24h)	EXP (LT)	EXP (24h)	EXP (LT)	EXP (24h)	EXP (LT)
EXP (LT)	0.536		0.023		0.390		n.d.	
STAT (LT)	0.536	0.940	**0.002**	**0.0002**	0.014	**0.0004**	n.d.	**0.006**

b)

	Nitrosopumilus sp. CCS1	
	ASW	ASW-HEPES
ASW-HEPES	0.021	
NSW	0.279	0.167

c)

	Nitrospina sp. Nb-3				*N. marina* Nb-295
	ASW (1µM, 15°C)	NSW (1µM, 15°C)	ASW (1mM, 25°C)	ASW+NH_4^+ (1mM, 25°C)	ASW (1mM, 25°C)
NSW (1µM, 15°C)	**0.006**				n.d.
ASW (1mM, 25°C)	0.192	**0.006**			
ASW+NH_4^+ (1mM, 25°C)	**0.007**	**0.008**	**0.006**		n.d.
ASW+trp (1mM, 25°C)	**0.008**	0.203	**0.009**	0.028	**0.003**

Table S4. Comparison of estimates of global dark ocean DIC fixation fueled by ammonia and nitrite oxidation

Nitrification-fueled global dark ocean DIC fixation ($Pg\ C\ y^{-1}$)	Reference
0.13 (NH_3 oxidation: 0.095; NO_2^- oxidation: 0.037)	this study
0.15 (NH_3 oxidation: 0.12; NO_2^- oxidation: 0.031)	(Zakem et al. 2018)
0.15-0.2 (NH_3 oxidation: 0.11-0.14; NO_2^- oxidation: 0.036-0.06)	(Zhang et al. 2020)
0.11 (NH_3 oxidation only)	(Middelburg 2011)
0.4 (NH_3 oxidation only)	(Herndl et al. 2005; Wuchter et al. 2006)
1 (NO_2^- oxidation only)	(Pachiadaki et al. 2017)

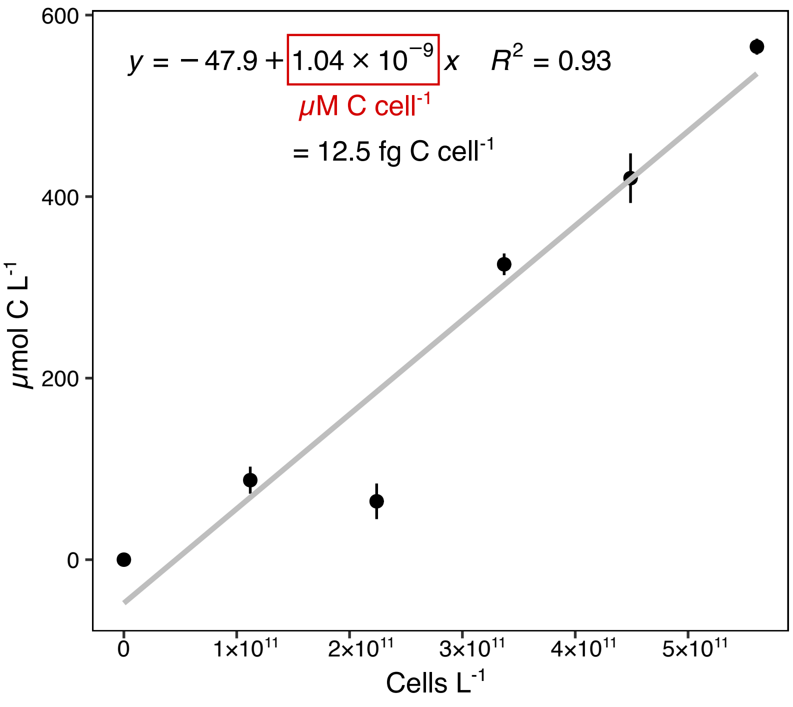

Fig. S1 Dilution series of concentrated cells to determine the cellular C content of *Nitrosopumilus* sp. CCS1

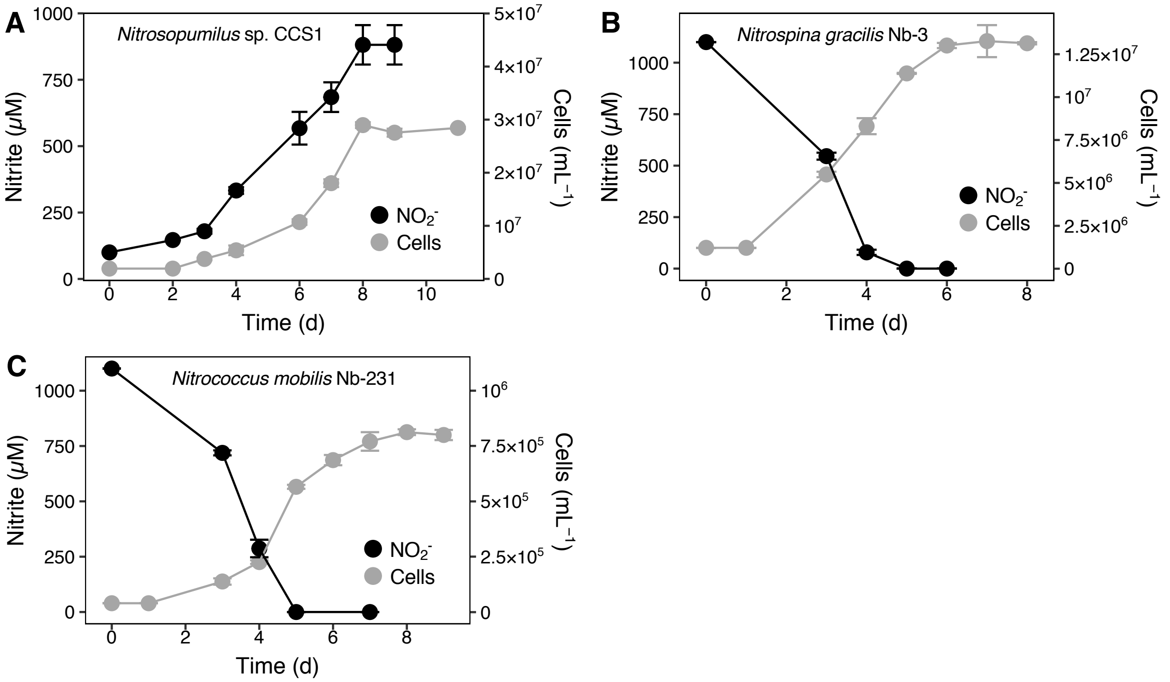

Fig. S2 Nitrite and cell concentrations of *Nitrosopumilus* sp. CCS1 (A), *Nitrospina* sp. Nb-3 (B) and *Nitrococcus mobilis* Nb-231 (C).

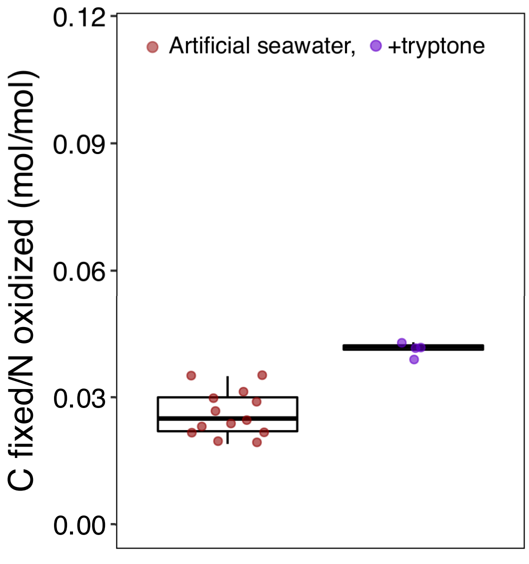

Fig. S3. DIC fixation yield of *N. marina* Nb-295 grown in artificial seawater medium with and without addition of tryptone.

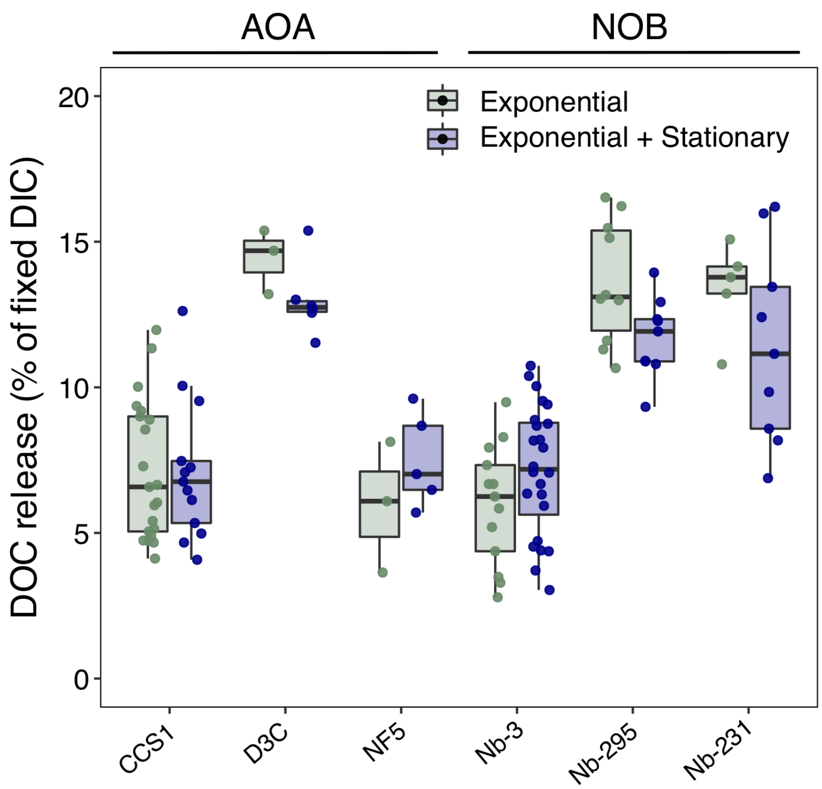

Fig. S4. DOC release (as a proportion of fixed DIC) of marine nitrifiers during different growth phases.

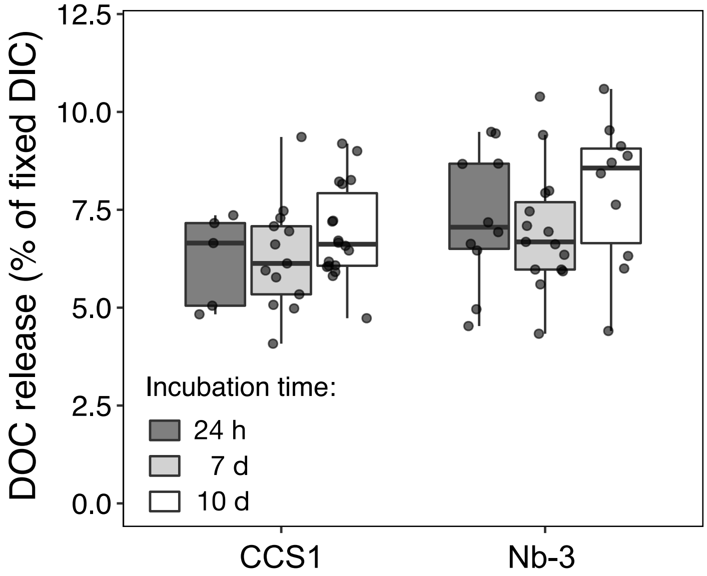

Fig. S5. DOC release (as a proportion of fixed DIC) of *Nitrosopumilus* sp. CCS1 and *Nitrospina* sp. Nb-3 at varying incubation times.

References

Bayer, B., J. Vojvoda, P. Offre, and others. 2016. Physiological and genomic characterization of two novel marine thaumarchaeal strains indicates niche differentiation. ISME Journal **10**: 1051–1063. doi:10.1038/ismej.2015.200

Bayer, B., J. Vojvoda, T. Reinthaler, C. Reyes, M. Pinto, and G. J. Herndl. 2019. Nitrosopumilus adriaticus sp. nov. and Nitrosopumilus piranensis sp. nov., two ammonia-oxidizing archaea from the Adriatic Sea and members of the class Nitrososphaeria. International Journal of Systematic and Evolutionary Microbiology **7**: 1892–1902. doi:10.1099/ijsem.0.003360

Carini, P., C. L. Dupont, and A. E. Santoro. 2018. Patterns of thaumarchaeal gene expression in culture and diverse marine environments. Environmental Microbiology **20**: 2112–2124. doi:10.1111/1462-2920.14107

Herndl, G. J., T. Reinthaler, E. Teira, H. van Aken, C. Veth, A. Pernthaler, and J. Pernthaler. 2005. Contribution of Archaea to total prokaryotic production in the deep Atlantic Ocean. Applied and environmental microbiology **71**: 2303–2309. doi:10.1128/AEM.71.5.2303

Koops, H.-P., B. Böttcher, U. C. Möller, a Pommerening-Röser, and G. Stehr. 1991. Classification of eight new species of ammonia-oxidizing bacteria. Journal of General Microbiology **137**: 1689–1699.

Lücker, S., B. Nowka, T. Rattei, E. Spieck, and H. Daims. 2013. The genome of Nitrospina gracilis illuminates the metabolism and evolution of the major marine nitrite oxidizer. Frontiers in Microbiology **4**: 27. doi:10.3389/fmicb.2013.00027

Middelburg, J. J. 2011. Chemoautotrophy in the ocean. Geophysical Research Letters **38**: 94–97. doi:10.1029/2011GL049725

Pachiadaki, M. G., E. Sintes, K. Bergauer, J. M. Brown, N. R. Record, B. K. Swan, and M. E. Mathyer. 2017. Major role of nitrite-oxidizing bacteria in dark ocean carbon fixation. Science **1051**: 1046–1051.

Santoro, A. E., and K. L. Casciotti. 2011. Enrichment and characterization of ammonia-oxidizing archaea from the open ocean: Phylogeny, physiology and stable isotope fractionation. ISME Journal **5**: 1796–1808. doi:10.1038/ismej.2011.58

Varela, M. M., H. M. van Aken, E. Sintes, T. Reinthaler, and G. J. Herndl. 2011. Contribution of Crenarchaeota and Bacteria to autotrophy in the North Atlantic interior. Environ Microbiol **13**: 1524–1533. doi:10.1111/j.1462-2920.2011.02457.x

Watson, S. W., E. Bock, F. W. Valois, J. B. Waterbury, and U. Schlosser. 1986. Nitrospira marina gen. nov. sp. nov.: a chemolithotrophic nitrite-oxidizing bacterium. Archives of Microbiology **144**: 1–7. doi:10.1007/BF00454947

Watson, S. W., and M. Mandel. 1971. Comparison of the morphology and deoxyribonucleic acid composition of 27 strains of nitrifying bacteria. Journal of Bacteriology **107**: 563–569. doi:10.1128/jb.107.2.563-569.1971

Watson, S. W., and J. B. Waterbury. 1971. Characteristics of two marine nitrite oxidizing bacteria, Nitrospina gracilis nov. gen. nov. sp. and Nitrococcus mobilis nov. gen. nov. sp. Archiv für Mikrobiologie **77**: 203–230. doi:10.1007/BF00408114

Wuchter, C., B. Abbas, M. J. L. Coolen, and others. 2006. Archaeal nitrification in the ocean. Proc. Natl. Acad. Sci. USA **103**: 12317–12322.

Zakem, E. J., A. Al-Haj, M. J. Church, and others. 2018. Ecological control of nitrite in the upper ocean. Nature Communications **9**. doi:10.1038/s41467-018-03553-w

Zhang, Y., W. Qin, L. Hou, and others. 2020. Nitrifier adaptation to low energy flux controls inventory of reduced nitrogen in the dark ocean. Proceedings of the National Academy of Sciences of the United States of America **117**: 4823–4830. doi:10.1073/pnas.1912367117
